# Composition Dependency of the Flory–Huggins Interaction Parameter in Drug–Polymer Phase Behavior

**DOI:** 10.3390/pharmaceutics15122650

**Published:** 2023-11-21

**Authors:** Jana Klueppelberg, Ulrich A. Handge, Markus Thommes, Judith Winck

**Affiliations:** 1 Laboratory of Solids Process Engineering, Department of Biochemical and Chemical Engineering, TU Dortmund University, Emil-Figge-Street 68, 44227 Dortmund, Germany; jana.klueppelberg@tu-dortmund.de (J.K.); markus.thommes@tu-dortmund.de (M.T.); 2 Chair of Plastics Technology, Department of Mechanical Engineering, TU Dortmund University, Leonhard-Euler-Street 5, 44227 Dortmund, Germany; ulrich.handge@tu-dortmund.de

**Keywords:** formulation design, phase diagrams, solubility, glass transition, Flory–Huggins theory

## Abstract

An innovative strategy to address recent challenges in the oral administration of poorly soluble drugs is the formulation of amorphous solid dispersions (ASDs), where the drug is dissolved in a highly soluble carrier polymer. Therefore, special knowledge of the drug–polymer phase behavior is essential for an effective product and process design, accelerating the introduction of novel efficacious ASD products. Flory–Huggins theory can be applied to model solubility temperatures of crystalline drugs in carrier polymers over the drug fraction. However, predicted solubility temperatures lack accuracy in cases of strong drug/polymer interactions that are not represented in the Flory–Huggins lattice model. Within this study, a modeling strategy is proposed to improve the predictive power through an extension of the Flory–Huggins interaction parameter by a correlation with the drug fraction. Therefore, the composition dependency of the Flory–Huggins interaction parameter was evaluated experimentally for various drug–polymer formulations that cover a wide variety of drug and polymer characteristics regarding molecular weights, glass transition temperatures and melting temperatures, as well as drug–polymer interactions of different strengths and effects. The extended model was successfully approved for nine exemplary ASD formulations containing the drugs acetaminophen, itraconazole, and griseofulvine, as well as the following polymers: basic butylated methacrylate copolymer, Soluplus^®^, and vinylpyrrolidone/vinyl acetate copolymer. A high correlation between the predicted solubility temperatures and experimental and literature data was found, particularly at low drug fractions, since the model accounts for composition dependent drug–polymer interactions.

## 1. Introduction

The development and optimization of active pharmaceutical ingredients significantly contribute to finding an effective treatment for a wide variety of diseases, with a simultaneous reduction in side effects. Accordingly, the complexity of the drug molecules often increases, which can be accompanied by hydrophobic properties and an associated low water solubility [1,2]. However, when a drug is administered orally, it is in most cases essential that a specific amount of the drug enters the systemic circulation of the body via the gastrointestinal tract to achieve a sufficiently high bioavailability [3].

To increase the bioavailability of the active ingredients concerned, a wide variety of process engineering approaches have already been reported, such as reducing the particle size or using additives [2,4]. Another method is the formulation of an amorphous solid dispersion (ASD), where the drug is molecularly dispersed in a water-soluble amorphous carrier polymer [5]. This approach has been used to significantly increase the bioavailability in several studies, both in vitro and in vivo [1]. The strategy behind this method is based on the higher free energy of the amorphous form of the drug, which acts as a driving force for the increased water solubility and dissolution and absorption rates [6]. However, since this form of the drug is thermodynamically unstable, it must be kinetically stabilized using an amorphous polymer matrix [7].

Depending on the combination and composition of the drug and the polymer, ASDs are characterized as metastable. Since their energetic state is still higher than that of a crystalline drug, a tendency to phase-separate and recrystallize is observed. In order to evaluate suitable drug–polymer combinations as well as the optimum process conditions during manufacturing, a thorough knowledge of the phase behavior of the binary system is essential. This phase behavior can be evaluated based on phase diagrams, which are defined by the solubility temperature of the crystalline drug in the polymer and the glass transition temperature of the ASD [8].

Various approaches have been utilized to predict the course of the solubility temperature and construct the phase diagrams of ASD formulations. They are primarily classified as computational or analytical methods, whereby computational methods such as molecular dynamics (MD) simulations are defined by a numerical solution of Newton’s equation of motion [9]. MD simulations have been applied to calculate molecular-level drug–polymer interactions [10] and, based on this, to predict the miscibility and phase behavior of ASD formulations [11]. However, the use of computational methods for the construction of ASD phase diagrams is still sporadic, which is attributed to the complexity of these advanced computational models. Thus, a broad basis of experimentally validated studies on a huge variety of drug–polymer formulations is currently missing [9].

**Figure 1:**
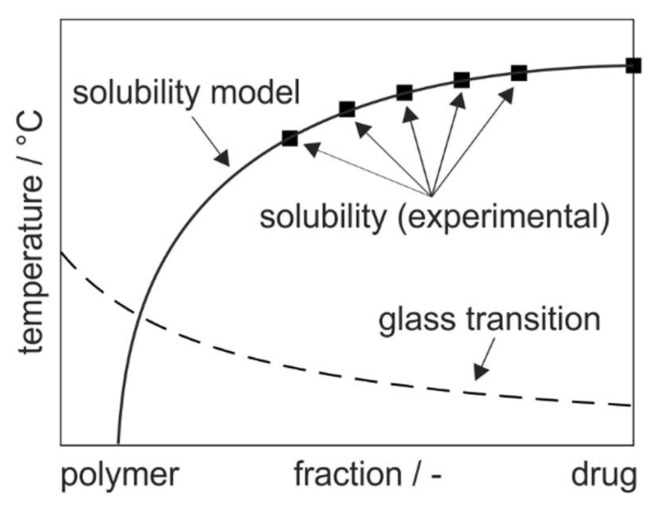
Schematic phase diagram of drug–polymer dispersions with the modeled solubility line (solid line) based on measured solubility temperatures of the crystalline drug in the polymer (symbols) and glass transition temperature of ASDs.

The phase diagrams of ASD formulations are usually constructed by measuring the solubility temperatures as depressed melting points of the crystalline drug in the polymer at specific drug weight fractions and afterwards modeling the solubility line over the whole range of compositions (Figure 1) [9]. Therefore, the depressed melting points are evaluated at high drug loadings, since calorimetric measurements at small drug loadings are limited by the slow dissolution near the glass transition temperature [12]. As a result of the wide range of extrapolation to lower drug loadings that is relevant for ASD production, an accurate prediction of the solubility temperatures is challenging [13].

Various modeling approaches are available to predict the course of the solubility line, from just empirical models to physically based and thermodynamic ones [14]. Empirical models that are solely based on the fitting of data points might suffer from the limited availability of experimental solubility data in the range of low-drug-weight fractions. The empirical model by Kyeremateng et al. was proposed in 2014, and within their study it was utilized to predict the solubility of the drugs naproxen, ibuprofen, itraconazole, acetaminophen, ibuprofen sodium, and nifedipine in the polymers vinylpyrrolidone/vinyl acetate copolymer and Soluplus^®^ [12]. Stability studies of two of the formulations containing ibuprofen and naproxen, as well as Soluplus^®^, were conducted to justify the validity of their modeling approach below the drug weight fractions that are accessible through calorimetric measurements.

Predicting solubility temperatures based on thermodynamic models requires a higher experimental effort to access pure components’ parameters in addition to the calorimetric data for the melting point depression of the crystalline drug in the polymer. However, these models offer a higher accuracy when predicting the solubility temperatures in a range that is not accessible through experiments, because the course of the solubility line is predefined by the previously measured material parameters. Thermodynamic models that are approved to describe the solubility of crystalline drugs in polymers are the Perturbed-Chain Statistical Associating Fluid Theory (PC–SAFT) and the Flory–Huggins model. PC–SAFT is an equation of state that is utilized to describe various material systems and is successfully applied to ASD formulations [8,15,16]. Because of the high number of PC–SAFT material parameters that have to be determined beforehand, the predicted solubilities show a precise correlation with experimental stability data [13]. These PC–SAFT parameters are the five pure-component parameters, namely, the segment number, the segment diameter, the dispersion-energy parameter, the association-energy parameter, and the association-volume parameter, as well as the number of association sites, the binary interaction parameter, the material density, and the molecular weight. However, applying the Flory–Huggins theory requires less experimental effort since, in addition to the calorimetric data, only the material densities and molecular weights of the components are evaluated.

The Flory–Huggins theory was initially developed by Flory [17] and Huggins [18] to describe the thermodynamics of polymer solutions. Based on a lattice model, the free energy of mixing is derived. It comprises an entropic contribution that represents the polymer chain configuration in the lattice and an enthalpic contribution that accounts for species interaction depending on the Flory–Huggins interaction parameter. The interaction parameter describes the strength of repulsive and attractive interactions between the different species which leads to a characteristic change of the solubility temperatures of the mixtures over the composition. Meanwhile, the Flory–Huggins approach is extensively applied to model solubility temperatures in ASD phase diagrams based on the melting point depression method [19]. Here, the melting point depression obtained by calorimetric measurements at higher drug weight fractions is correlated with the Flory–Huggins interaction parameter, which, again, is utilized to predict the solubility line over the whole compositional range of the ASD up to low-drug-weight fractions. However, when calculating one constant interaction parameter, the prediction of solubility temperatures in the range of low-drug-weight fractions where no experimental solubility data from calorimetric measurements are available lacks accuracy for some ASD formulations [20,21,22]. This is due to the complex dependence between the drug–polymer interactions and the temperature and the composition, as stated in the literature [23,24,25], which is not represented in the model and leads to inaccuracies in the extrapolation of the solubility temperature to lower drug weight fractions beyond the experimentally accessed range.

In this study, the composition dependency of the Flory–Huggins interaction parameter is investigated systematically for nine drug–polymer formulations. Thereof, the findings are transferred to propose a novel approach of extending the Flory–Huggins theory by a composition-dependent term to improve the accuracy when predicting solubility temperatures over the whole compositional range of drug–polymer formulations, and particularly at lower drug weight fractions. The knowledge of these precisely predicted solubility temperatures is highly relevant for the design and manufacturing of ASD formulations as well as the assessment of their stability.

## 2. Materials and Methods

### 2.1. Materials

**Figure 2:**
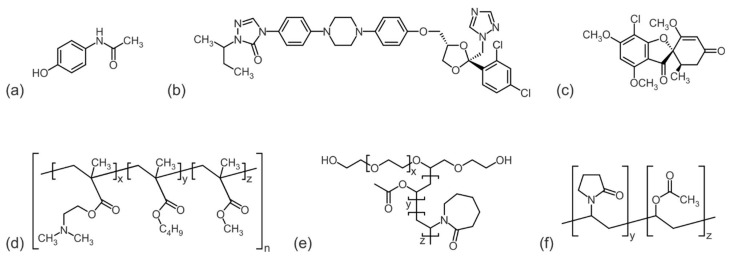
Molecular structure of drugs: (**a**) acetaminophen, (**b**) itraconazole, (**c**) griseofulvine and polymers, (**d**) basic butylated methacrylate copolymer, (**e**) Soluplus^®^, and (**f**) vinylpyrrolidone/vinyl acetate copolymer.

Three model drugs were selected to prepare ASD formulations with three commonly used excipient polymers, respectively (Figure 2).

The drugs acetaminophen (ACE) (Caelo, Caesar & Loretz GmbH, Hilden, Germany), itraconazole (ITR) (BASF SE, Ludwigshafen, Germany) and griseofulvin (GRI) (Hawkins, Roseville, MN, USA) cover a broad spectrum of molecular weight (Table 1). Furthermore, the polymers basic butylated methacrylate copolymer (bBMA) EUDRAGIT^®^ E PO (Evonik Industries AG, Darmstadt, Germany), vinylpyrrolidone/vinyl acetate copolymer (PVPVA) Kollidon^®^ VA64 (BASF SE, Ludwigshafen, Germany), and Soluplus^®^ (SOL) (BASF SE, Ludwigshafen, Germany), an ethylene glycol/vinylcaprolactam/vinyl acetate copolymer, were selected.

### 2.2. Differential Scanning Calorimetry

Calorimetric measurements were performed to determine thermal properties of drugs and polymers as well as to construct phase diagrams of ASD formulations according to the melting point depression method. In both cases, differential scanning calorimetry (DSC) was applied using a Q2000 heat flux DSC (TA Instruments, New Castle, DE, USA) that was purged with 50 mL/min nitrogen. For each measurement, between 3 and 7 mg of the sample was placed in an aluminum pan and sealed with a perforated lid.

A two-step heating protocol was applied to evaluate the glass transition and melting temperatures of the pure substances. In the first heating cycle, the melting temperatures and enthalpy were analyzed using a heating rate of 10 °C/min. The glass transition temperatures were measured in the second heating cycle in order to eliminate the thermal and mechanical history of the samples. Here, a heating rate of 20 °C/min was chosen and the glass transition temperatures were determined according to the inflection point method [33].

The phase diagrams of ASD formulations were constructed based on calorimetric measurements according to the melting point depression method, where the dissolution temperatures of physical drug–polymer mixtures are evaluated [22]. Therefore, the dissolution endpoints of milled physical mixtures were determined at different heating rates and extrapolated to a zero heating rate to detect the equilibrium solubility temperature of a specific drug weight fraction [34]. In this study, the samples were scanned with heating rates of 3 °C/min, 5 °C/min, and 10 °C/min. The dissolution was evaluated in the first heating circle and linearly extrapolated to a zero heating rate (0 °C/min). This was followed by a second heating circle at a heating rate of 20 °C/min to determine the glass transition temperatures of the ASD formulations. Before the calorimetric measurements, the physical mixtures were ground at 50 Hz for 9 min in three cycles using a Pulverisette 23 ball mill (Fritsch, Idar-Oberstein, Germany). Since ITR tended to aggregate during ball milling, the sample preparation was adopted for the formulations containing ITR. In these cases, a SPEX CertiPrep 6850 cryomill (SPEX CertiPrep, Metuchen, NJ, USA) was utilized for grinding at 10 Hz for 16 min in 8 circles.

### 2.3. Modeling of Phase Diagrams

The solubility lines of the phase diagrams were constructed on the basis of the melting point depression method according to different modeling approaches. The first approach is an empirical equation proposed by Kyeremateng et al. [12].

(1) $$T_{s,ASD}=-A\cdot exp\left(-100\cdot b\cdot w_{drug}\right)+T_{m,drug}+C$$

The parameters A and b are applied to fit the solubility curve $T_{s,ASD}$ to the experimentally determined equilibrium solubility temperatures, measured by DSC at various drug weight fractions $w_{drug}$ (g/g). The parameter b was fixed to 0.05 as proposed.

Furthermore, the Flory–Huggins model was utilized [17,18].

(2) $$\frac{1}{T_{s,ASD}}-\frac{1}{T_{m,drug}}=-\frac{R}{\Delta h_{m,drug}}\left[ln\varphi_{drug}+\left(1-\frac{1}{m}\right)\varphi_{polymer}+\chi {\cdot \varphi }_{polymer}^{2}\right]$$

The interaction parameter χ is fitted to the experimental data, considering the solubility temperature $T_{s,ASD}$ at a specific volume fraction φ. $T_{m,drug}$ and $\Delta h_{m,drug}$ are melting temperature and enthalpy of the drug, respectively, and m is the ratio of the polymer to drug molar volume.

(3) $$m=\frac{M_{w,polymer}}{M_{w,drug}}\cdot \frac{\rho_{drug}}{\rho_{polymer}}$$

The composition dependence of the glass transition temperatures of ASD formulations was modeled using the Gordon–Taylor equation [35].

(4) $$T_{g,ASD}=\frac{w_{polymer}\cdot T_{g,polymer}+k\cdot w_{drug}\cdot T_{g,drug}}{w_{polymer}+k\cdot w_{drug}}$$

Based on the pure components glass transition temperatures $T_{g,drug}$ and $T_{g,polymer}$, the glass transition temperature of the mixture $T_{g,ASD}$ is estimated depending on the respective drug weight fraction $w_{drug}$. The Gordon–Taylor constant k is calculated from the densities of drug and polymer as well as the glass transition temperatures of the pure components according to the Simha–Boyer rule [36,37].

(5) $$k=\frac{T_{g,polymer}}{T_{g,drug}}\cdot \frac{\rho_{polymer}}{\rho_{drug}}$$

Parameter fitting for all phase diagram modeling approaches was performed with MATLAB^®^ (The MathWorks, Inc., Natick, MA, USA) based on a least-squares solver.

## 3. Results and Discussion

### 3.1. Composition Dependency of the Flory–Huggins Interaction Parameter

A common procedure for modeling the solubility line of a drug–polymer phase diagram based on the Flory–Huggins interaction parameter is to fit one constant interaction parameter to the experimental data of the melting point depression experiments. This is implemented by rearranging Equation (2) to a linear equation with the slope χ, which can be easily determined by plotting A (Equation (6), left) over B ($\varphi_{polymer}^{2}$) [34].

(6) $$\begin{array}{c} -\frac{\Delta h_{m,drug}}{R}\left(\frac{1}{T_{s,ASD}}-\frac{1}{T_{m,drug}}\right)-\left[ln\varphi_{drug}+\left(1-\frac{1}{m}\right)\varphi_{polymer}\right]=\chi \cdot \varphi_{polymer}^{2}\\ A=\chi \cdot B \end{array}$$

Usually, for the determination of the Flory–Huggins interaction parameter, a compositional range is considered that contains far over 50 wt% of drug so that a linear correlation between A and B was found [32,38]. This means that there was a constant Flory–Huggins interaction parameter across this specific drug loading range. However, it is known that the Flory–Huggins interaction parameter can depend on the composition [39].

**Figure 3:**
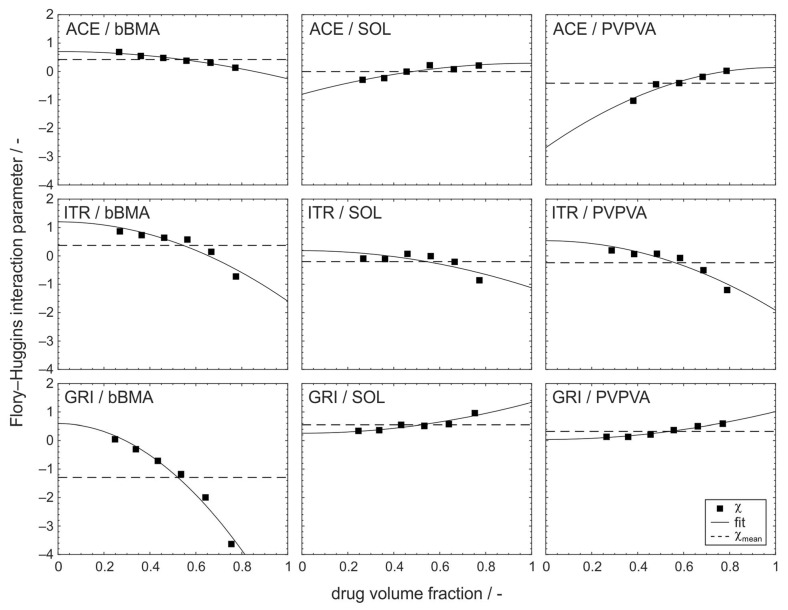
Flory–Huggins interaction parameter depending on the drug volume fraction of solid dispersions containing the drugs acetaminophen (**top**), itraconazole (**middle**), griseofulvine (**bottom**), and the polymers basic butylated methacrylate copolymer (**left**), Soluplus^®^ (**middle**), and polyvinylpyrrolidone/vinyl acetate copolymer (**right**).

In this study, the composition dependency of the Flory–Huggins interaction parameter was evaluated for nine drug–polymer formulations in the drug loading range between 30 wt% and 80 wt% (Figure 3). It is clear from the diagrams that for all nine formulations, the assumption of a constant interaction parameter $\chi_{mean}$ (dashed line) over the entire compositional range was not applicable. Instead, the interaction parameter followed a quadratic relationship with the volume fraction of the drug φ (solid line).

(7) $$\chi \left(\varphi \right)=\chi_{1}+\chi_{2}\cdot {\left(\varphi -\chi_{3}\right)}^{2}$$

This empirical model considers three parameters, whereby the $\chi_{1}$ parameter has the same physical meaning as the Flory–Huggins parameter and the constant interaction parameter $\chi_{mean}$ as discussed before. It can be seen as the intercept of the function (Figure 3, solid lines) describing the magnitude of the drug–polymer interaction at infinite dilution. The second parameter $\chi_{2}$ captures the gradient in molecular interaction with respect to volume fraction. This parameter is the key and the novelty in this study and extends the conventional Flory–Huggins model by a volume fraction dependency. Due to the quadratic nature of the model, the third parameter $\chi_{3}$ exists just at 0 and 1, basically as a Boolean parameter. When $\chi_{3}=0$, the absolute value of the gradient $\chi_{2}$ is smaller at φ=0, whereas at $\chi_{3}=1$, the absolute value of the gradient $\chi_{2}$ is smaller at φ=1. This parameter can be eliminated from the model when sorting the data where the stronger interaction is always at φ=1. However, this might lead to some confusion in visualization (Figure 3) when inverting the meaning of the φ-axis for some graphs. Therefore, this parameter was implemented. Unfortunately, if $\chi_{3}=1$, the $\chi_{2}$ parameter needs to be multiplied by −1 in order to have the correct physical meaning (stronger intensification of higher molecular interaction with volume fraction at lower value).

In summary, the new model uses just two parameters, where $\chi_{1}$ has the same physical meaning as the conventional Flory–Huggins parameter whereas $\chi_{2}$ considers the interaction dependency from the volume fraction.

This correlation of the interaction parameter implies that the intensity and direction of the interactions between polymer and drug are variable with the amount of drug in the formulation, as shown in Figure 3. In some of the formulations considered here, there is even a change in the sign of the interaction parameter with the drug volume fraction. This indicates a change in dominance in the interaction, related to cohesiveness or adhesiveness. For the formulations containing the drugs ACE or GRI and the polymers SOL or PVPVA, respectively, the interaction parameter becomes smaller for a decreasing drug volume fraction, corresponding to an increase in the attractive interaction. The other formulations show an opposite relation where the attractive interaction decreases with the drug volume fraction.

### 3.2. Improved Modeling of Phase Diagrams Based on the Flory–Huggins Interaction Parameter

The challenge of applying the Flory–Huggins theory for the construction of phase diagrams is the underlying assumption of statistically distributed polymer segments and drug molecules when mixing the components, which is accompanied by the assumption of a constant Flory–Huggins interaction parameter over the whole compositional range. Since most active ingredients have a protonic character, i.e., can absorb or release protons, real mixing processes lead to the formation of hydrogen bonds, among other things. These composition-specific drug–polymer interactions are not sufficiently represented by the model because the assumption of randomly distributed drug molecules next to polymer segments during mixing is, in these cases, not fulfilled, especially for strongly interacting drug–polymer systems. For this reason, the solubility of the system can be underestimated, which means that in reality the phase equilibrium is at lower temperatures than determined experimentally [14,40]. Assuming a constant Flory–Huggins interaction parameter over the whole compositional range can thus lead to an inaccurate prediction of phase diagrams in comparison to other models for constructing solubility curves, such as PC–SAFT [13].

In order to account for the composition dependency of the Flory–Huggins interaction parameter when constructing solubility curves for drug–polymer phase diagrams, the Flory–Huggins Equation (2) is extended to the observed quadratic correlation between χ and φ (Equation (7)), called extended Flory–Huggins (xFH) within this study.

(8) $$\begin{array}{cc} \frac{1}{T_{s,ASD}}-\frac{1}{T_{m,drug}} & =-\frac{R}{\Delta h_{m,drug}}[ln\varphi_{drug}+\left(1-\frac{1}{m}\right)\varphi_{polymer}\\ \; & +\left(\chi_{1}+\chi_{2}\cdot {\left(\varphi -\chi_{3}\right)}^{2}\right){\cdot \varphi }_{polymer}^{2}] \end{array}$$

**Figure 4:**
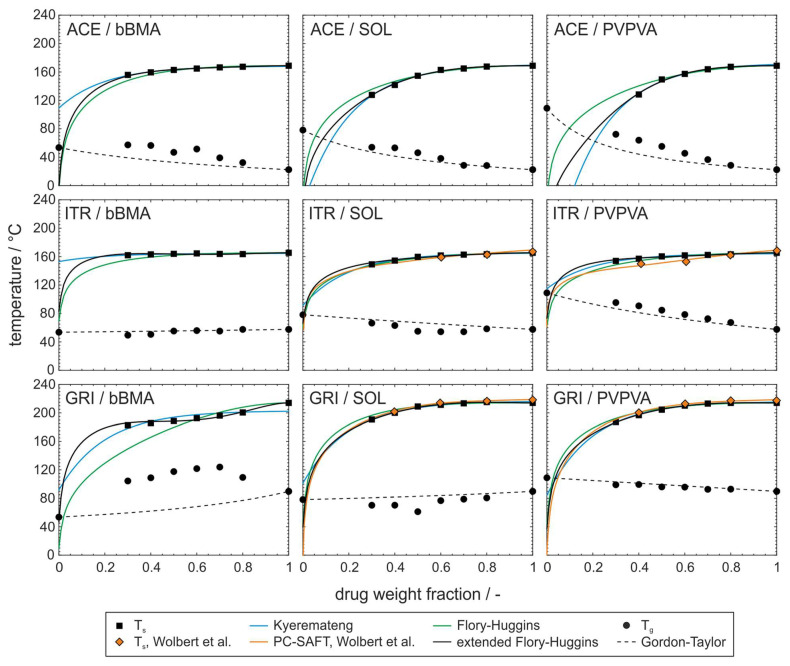
Phase behavior of solid dispersions containing the drugs acetaminophen (**top**), itraconazole (**middle**), griseofulvine (**bottom**), and the polymers basic butylated methacrylate copolymer (**left**), Soluplus^®^ (**middle**), and vinylpyrrolidone/vinyl acetate copolymer (**right**). The literature data from Wolbert et al. [27] are taken from their Supplementary Materials.

In Figure 4, phase diagrams for the nine investigated drug–polymer formulations are shown with their solubility curves that were constructed based on different modeling approaches. In actuality, the conventional Flory–Huggins, the extended Flory–Huggins, and the empirical model are compared. Additionally, the literature data of PC–SAFT from Wolbert et al. [27] are depicted for the formulations containing the drugs ITR or GRI and the polymers SOL or PVPVA. Furthermore, glass transition temperatures and the Gordon–Taylor model are given to describe them over the drug loading fraction.

The conventional Flory–Huggins model using a constant interaction parameter does not describe the experimental data of solubility temperatures accurately. Especially in the range of small drug loading, relevant for pharmaceutical applications, the results seem to deviate more and more from the experimentally determined values. It can be concluded that the model also fails in the extrapolation of solubility temperatures to drug weight fractions lower than the measured values. The conventional Flory–Huggins model deviates particularly at low drug weight fractions because at lower temperatures more drug–polymer interactions occur that are not represented in the modeling approach.

The comparative presentation shows that the phase equilibrium is described more accurately with the extended Flory–Huggins model, especially in the region of low-drug-weight fractions. This is also confirmed by a comparison with the established and extensively validated PC–SAFT model. However, when the experimental solubility data for the PC–SAFT modeling deviate from the solubility temperatures obtained in this study, as for the formulation ITR/PVPVA, the fitted solubility curves will accordingly not show a high correlation, either. Such deviations of experimentally determined solubility temperatures can be explained by differences in sample preparation or measuring procedure which could not be investigated further [41]. It is also clear that the extended Flory–Huggins approach can describe particularly inconsistent solubility equilibria such as found for GRI/bBMA.

The empirical model deviates increasingly from the predicted phase behavior with decreasing drug loading. Compared to the other two models, the empirical model is not based on any physics, but is only approximated to the experimental data with two fitting parameters. For drug weight fractions where sufficient experimental data are available (>30 wt%), the empirical model can provide a good approximation of solubility temperatures. For a correct description when extrapolating into ranges for which there is no experimental basis, the accuracy is not sufficient.

A detailed observation of the glass transition temperatures shows that they are sometimes not well described by the Gordon–Taylor model. This may be due to the fact that the Gordon–Taylor approach assumes an ideally mixed phase and, for this reason, is only applicable to completely amorphous and single phase systems. However, in ASDs, due to the thermodynamic instability of the phase, diffusion processes over time can lead to the formation of clusters rich in active ingredients and polymers, which can result in local supersaturation. For drug loadings close to 100 wt%, the approach again matches the experimentally determined values much better, since the value approaches the glass transition of the pure drug. The deviation from the Gordon–Taylor model can accordingly be formulated as an indication of the lack of homogeneity of the phase and suitability of the formulation as an ASD. The following systems show a positive deviation from the Gordon–Taylor model and are ranked according to the degree of deviation: GRI/bBMA, ACE/bBMA, ACE/PVPVA, ACE/SOL, and ITR/PVPVA. A positive deviation is interpreted as an indication of strong attractive interactions [42]. The systems ordered by the intensity of the negative deviation from the model have, according to theory, comparatively weak intermolecular interactions: ITR/SOL and GRI/SOL. For these systems, the correlation may be an indication of the lack of kinetic stability of the phase. On the other hand, the systems GRI/PVPVA and ITR/bBMA show a good approximation with the model and, according to the described theory, can form a homogeneous amorphous phase, and with their kinetic stability are suitable for the formulation as ASD.

Since a negative interaction parameter of the Flory–Huggins theory is also correlated with attractive interactions and, in magnitude, with the strength of the interaction, the comparison with the systems that have a negative interaction parameter (Table 2) seems reasonable. These include GRI/bBMA, ACE/PVPVA, ITR/PVPVA, ITR/SOL, and ACE/SOL. Therefore, attractive interactions were detected by both models, Gordon–Taylor and Flory–Huggins, except for the formulations ACE/bBMA and ITR/SOL.

### 3.3. Adoption to ASD Manufacturing Processes

**Figure 5:**
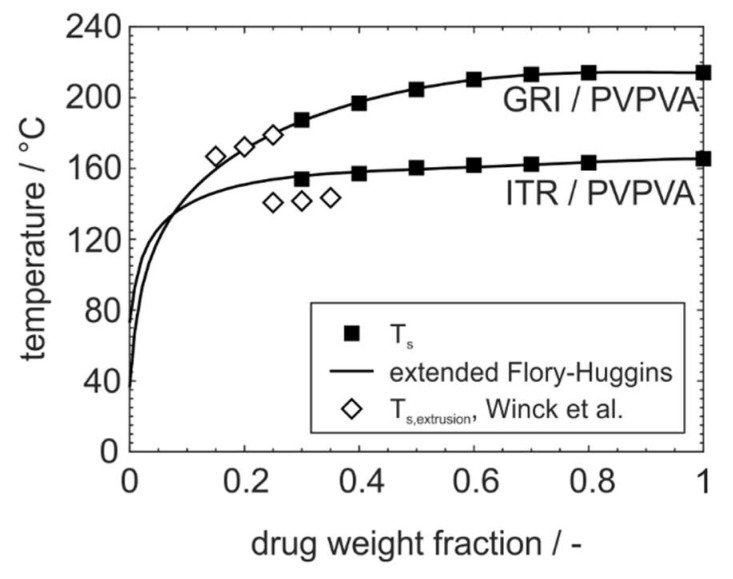
Comparison of solubility temperatures modeled with extended Flory–Huggins within this study and dissolution temperatures obtained by hot melt extrusion for the formulations ITR/PVPVA and GRI/PVPVA at a residence time of 10 min from Winck et al. [43].

In order to evaluate the solubility temperatures predicted by the extended Flory–Huggins model for small drug loadings relevant for ASD formulations, a comparison with the literature data from Winck et al. [43] is shown in Figure 5. In their study, physical drug–polymer mixtures were processed at various drug weight fractions and temperatures using a mini extruder in order to specify the temperature at which the drug was completely dissolved in the polymer at a residence time of 10 min. For this purpose, the dissolution endpoints were detected using in-line UV–Vis spectroscopy.

The comparison of dissolution endpoints in extrusion with the solubility temperatures predicted by the Flory–Huggins model in this study reveals some deviations. For the formulation GRI/PVPVA, the dissolution temperatures from extrusion at 20 wt% and 25 wt% drug loading perfectly fit the predicted solubility line, whereas at 15 wt%, the dissolution endpoint in extrusion was detected at a temperature slightly above the predicted solubility temperature. For the formulation GRI/PVPVA, the slope of the solubility line corresponds the course of the dissolution endpoints in extrusion. However, the solubility line is shifted to higher temperatures in comparison to the dissolution endpoints. Here, it seems reasonable that the drug particle size could not be reduced sufficiently during milling to eliminate all kinetic effects in DSC melting point depression measurements [44]. In extrusion, an equilibrium can be reached straightforwardly due to the superimposed mechanical stresses that are applied on the materials [45]. Furthermore, differing resolutions of the spectroscopic and thermal measurements should be considered [46].

## 4. Conclusions

A novel method was proposed to account for the composition dependency of the Flory–Huggins interaction parameter when constructing drug–polymer phase diagrams for ASD processing based on the melting point depression method. Therefore, the quadratic relation that was detected experimentally between the Flory–Huggins interaction parameter and the drug volume fraction was implemented into the Flory–Huggins model, called extended Flory–Huggins (xFH). This method addresses the challenge to predict the solubility temperatures of crystalline drugs in polymers over the whole compositional range with sufficient accuracy. For the design of ASD formulations, the prediction of the range of lower drug weight fractions (<40 wt%) is of particular interest. However, this relevant range is not accessible through calorimetric measurements associated with the melting point depression method, since the solubility temperature is close to the glass transition temperature here, so the drug dissolution kinetics are too slow.

The extended model xFH was successfully approved for all nine drug–polymer formulations containing the drugs acetaminophen, itraconazole, and griseofulvine, as well as the polymers basic butylated methacrylate copolymer, Soluplus^®^, and vinylpyrrolidone/vinyl acetate copolymer that were investigated within this study. These nine formulations covered a wide variety of drug and polymer characteristics regarding molecular weight, glass transition temperature and melting temperature, as well as drug–polymer interactions of different strength and effect, which confirms the wide applicability of the proposed model. Experimental data points for the solubility temperatures of the crystalline drugs in the polymers were determined for 30, 40, 50, 60, 70, and 80 wt%, respectively. At higher drug weight fractions, the experimental data are well described by all considered models. However, in the compositional range relevant for ASD formulation design, large deviations between the different modeling approaches were detected. A higher correlation of xFH to the experimental solubility data and PC–SAFT [27] was found in comparison to both the conventional Flory–Huggins and the empirical model of Kyeremateng et al. [12]. Specifically, this high correlation of xFH at low drug fractions is essential for the successful prediction of solubility temperatures for ASD formulation design, since it was demonstrated that the lower correlation of the conventional Flory–Huggins model can result in a deviation of the predicted solubility temperature of more than 50 °C at drug weight fractions of 10 wt%, depending on the drug–polymer formulation. The deviation of the results of the conventional Flory–Huggins theory is explained by the consideration of one constant interaction parameter which does not account for the interactions between drug and polymer that are dependent on the composition, i.e., hydrogen bonding that contradicts the assumption of statistically distributed polymer segments and drug molecules. The empirical approach of Kyeremateng is not based on a physical model so that the fit of the solubility line is, in comparison to the other models, not forced to a specific material or material interaction parameters at low-drug-weight fractions. This can result in a low accuracy at lower drug weight fractions where no experimental data are available for the curve fitting.

## Figures and Tables

**Table 1:** Material data of drugs and polymers.

Substance	$M_{w}$ g/mol	$\rho_{true}$ $g/{cm}^{3}$	$\Delta h_{m}$ J/g	$T_{g}$ °C	$T_{m}$ °C
ACE	151.2 ^a^	1.29 ^b^	179.2	22.5	170.0
ITR	705.6 ^c^	1.27 ^d^	85.8	57.6	164.9
GRI	352.8 ^c^	1.42 ^e^	103.8	89.8	220.5
bBMA	47 000 ^f^	1.09 ^g^	–	53.6	–
SOL	118 000 ^h^	1.08 ^g^	–	78.2	–
PVPVA	57 500 ^h^	1.19 ^g^	–	109.0	–

^a^ Lehmkemper et al. [13], ^b^ Nair et al. [26], ^c^ Wolbert et al. [27], ^d^ Six et al. [28], ^e^ Zhou et al. [29], ^f^ Dos Santos et al. [30], ^g^ Gottschalk et al. [31], ^h^ Altamimi and Neau [32].

**Table 2:** Parameters of the empirical model, Flory–Huggins (FH), and composition-dependent Flory–Huggins (xFH).

Formulation	Empirical		FH	xFH		
	A	C	χ	$\chi_{1}$	$\chi_{2}$	$\chi_{3}$
ACE/bBMA	59.01	−0.5662	0.4210	0.7025	−0.9608	0
ACE/SOL	198.21	2.1004	−0.0047	0.2893	−1.0933	1
ACE/PVPVA	316.10	3.9999	−0.4130	0.1379	−2.8219	1
ITR/bBMA	11.65	−0.7288	0.3714	1.2006	−2.7956	0
ITR/SOL	73.99	−0.1340	−0.2013	0.1846	−1.3103	0
ITR/PVPVA	49.59	−0.9585	−0.2407	0.5347	−2.4495	0
GRI/bBMA	111.32	−11.0231	−1.2948	0.5949	−6.9489	0
GRI/SOL	115.47	2.6304	0.5522	0.2617	1.0764	0
GRI/PVPVA	131.77	1.9122	0.3252	0.0419	0.9730	0

## Data Availability

Data are contained within the article.

## References

[1] Schittny A., Huwyler J., Puchkov M. (2020). Mechanisms of increased bioavailability through amorphous solid dispersions: A review. Drug Deliv..

[2] Rodriguez-Aller M., Guillarme D., Veuthey J.-L., Gurny R. (2015). Strategies for formulating and delivering poorly water-soluble drugs. J. Drug Deliv. Sci. Technol..

[3] Vasconcelos T., Sarmento B., Costa P. (2007). Solid dispersions as strategy to improve oral bioavailability of poor water soluble drugs. Drug Discov. Today.

[4] Chu K.R., Lee E., Jeong S.H., Park E.-S. (2012). Effect of particle size on the dissolution behaviors of poorly water-soluble drugs. Arch. Pharm. Res..

[5] Bhujbal S.V., Mitra B., Jain U., Gong Y., Agrawal A., Karki S., Taylor L., Kumar S., Zhou Q. (2021). Pharmaceutical amorphous solid dispersion: A review of manufacturing strategies. Acta Pharm. Sin. B.

[6] Hancock B.C., Parks M. (2000). What is the true solubility advantage for amorphous pharmaceuticals?. Pharm. Res..

[7] Baghel S., Cathcart H., O’Reilly N.J. (2016). Polymeric amorphous solid dispersions: A review of amorphization, crystallization, stabilization, solid-state characterization, and aqueous solubilization of biopharmaceutical classification system class II drugs. J. Pharm. Sci..

[8] Prudic A., Ji Y., Sadowski G. (2014). Thermodynamic phase behavior of API/polymer solid dispersions. Mol. Pharm..

[9] Medarević D., Djuriš J., Barmpalexis P., Kachrimanis K., Ibrić S. (2019). Analytical and Computational Methods for the Estimation of Drug-Polymer Solubility and Miscibility in Solid Dispersions Development. Pharmaceutics.

[10] Barmpalexis P., Karagianni A., Katopodis K., Vardaka E., Kachrimanis K. (2019). Molecular modelling and simulation of fusion-based amorphous drug dispersions in polymer/plasticizer blends. Eur. J. Pharm. Sci..

[11] Walden D.M., Bundey Y., Jagarapu A., Antontsev V., Chakravarty K., Varshney J. (2021). Molecular Simulation and Statistical Learning Methods toward Predicting Drug-Polymer Amorphous Solid Dispersion Miscibility, Stability, and Formulation Design. Molecules.

[12] Kyeremateng S.O., Pudlas M., Woehrle G.H. (2014). A fast and reliable empirical approach for estimating solubility of crystalline drugs in polymers for hot melt extrusion formulations. J. Pharm. Sci..

[13] Lehmkemper K., Kyeremateng S.O., Heinzerling O., Degenhardt M., Sadowski G. (2017). Long-Term Physical Stability of PVP- and PVPVA-Amorphous Solid Dispersions. Mol. Pharm..

[14] Thakore S.D., Akhtar J., Jain R., Paudel A., Bansal A.K. (2021). Analytical and Computational Methods for the Determination of Drug-Polymer Solubility and Miscibility. Mol. Pharm..

[15] Prudic A., Kleetz T., Korf M., Ji Y., Sadowski G. (2014). Influence of copolymer composition on the phase behavior of solid dispersions. Mol. Pharm..

[16] Prudic A., Ji Y., Luebbert C., Sadowski G. (2015). Influence of humidity on the phase behavior of API/polymer formulations. Eur. J. Pharm. Biopharm..

[17] Flory P.J. (1941). Thermodynamics of High Polymer Solutions. J. Chem. Phys..

[18] Huggins M.L. (1942). Thermodynamic properties of solutions of long-chain compounds. Ann. N. Y. Acad. Sci..

[19] Marsac P.J., Shamblin S.L., Taylor L.S. (2006). Theoretical and practical approaches for prediction of drug-polymer miscibility and solubility. Pharm. Res..

[20] Paudel A., van Humbeeck J., van den Mooter G. (2010). Theoretical and experimental investigation on the solid solubility and miscibility of naproxen in poly(vinylpyrrolidone). Mol. Pharm..

[21] Wlodarski K., Sawicki W., Kozyra A., Tajber L. (2015). Physical stability of solid dispersions with respect to thermodynamic solubility of tadalafil in PVP-VA. Eur. J. Pharm. Biopharm..

[22] Sun Y., Tao J., Zhang G.G.Z., Yu L. (2010). Solubilities of crystalline drugs in polymers: An improved analytical method and comparison of solubilities of indomethacin and nifedipine in PVP, PVP/VA, and PVAc. J. Pharm. Sci..

[23] Koningsveld R., Stockmayer W.H., Nies E. (2008). Polymer Phase Diagrams: A Textbook.

[24] Wilkie C.A. (2014). Polymer Blends Handbook.

[25] Potter C.B., Davis M.T., Albadarin A.B., Walker G.M. (2018). Investigation of the Dependence of the Flory-Huggins Interaction Parameter on Temperature and Composition in a Drug-Polymer System. Mol. Pharm..

[26] Nair R., Nyamweya N., Gönen S., Martínez-Miranda L.J., Hoag S.W. (2001). Influence of various drugs on the glass transition temperature of poly(vinylpyrrolidone): A thermodynamic and spectroscopic investigation. Int. J. Pharm..

[27] Wolbert F., Fahrig I.-K., Gottschalk T., Luebbert C., Thommes M., Sadowski G. (2022). Factors influencing the crystallization-onset time of metastable ASDs. Pharmaceutics.

[28] Six K., Verreck G., Peeters J., Brewster M., van den Mooter G. (2004). Increased physical stability and improved dissolution properties of itraconazole, a class II drug, by solid dispersions that combine fast- and slow-dissolving polymers. J. Pharm. Sci..

[29] Zhou D., Zhang G.G.Z., Law D., Grant D.J.W., Schmitt E.A. (2008). Thermodynamics, molecular mobility and crystallization kinetics of amorphous griseofulvin. Mol. Pharm..

[30] Dos Santos J., Da Silva G.S., Velho M.C., Beck R.C.R. (2021). Eudragit^®^: A Versatile Family of Polymers for Hot Melt Extrusion and 3D Printing Processes in Pharmaceutics. Pharmaceutics.

[31] Gottschalk T., Özbay C., Feuerbach T., Thommes M. (2022). Predicting Throughput and Melt Temperature in Pharmaceutical Hot Melt Extrusion. Pharmaceutics.

[32] Altamimi M.A., Neau S.H. (2016). Use of the Flory–Huggins theory to predict the solubility of nifedipine and sulfamethoxazole in the triblock, graft copolymer Soluplus. Drug Dev. Ind. Pharm..

[33] Höhne G., Hemminger W., Flammersheim H.-J. (2003). Differential Scanning Calorimetry: An Introduction for Practitioners.

[34] Moseson D.E., Taylor L.S. (2018). The application of temperature-composition phase diagrams for hot melt extrusion processing of amorphous solid dispersions to prevent residual crystallinity. Int. J. Pharm..

[35] Gordon M., Taylor J.S. (1952). Ideal copolymers and the second-order transitions of synthetic rubbers. i. non-crystalline copolymers. J. Appl. Chem..

[36] Simha R., Boyer R.F. (1962). On a General Relation Involving the Glass Temperature and Coefficients of Expansion of Polymers. J. Chem. Phys..

[37] Katkov I.I., Levine F. (2004). Prediction of the glass transition temperature of water solutions: Comparison of different models. Cryobiology.

[38] Hu Z., Xu P., Ashour E.A., Repka M.A. (2022). Prediction and Construction of Drug-Polymer Binary System Thermodynamic Phase Diagram in Amorphous Solid Dispersions (ASDs). AAPS PharmSciTech.

[39] Chaudhari M.I., Pratt L.R., Paulaitis M.E. (2014). Concentration dependence of the Flory-Huggins interaction parameter in aqueous solutions of capped PEO chains. J. Chem. Phys..

[40] Anderson B.D. (2018). Predicting Solubility/Miscibility in Amorphous Dispersions: It Is Time to Move Beyond Regular Solution Theories. J. Pharm. Sci..

[41] Ehrenstein G.W., Riedel G., Trawiel P. (2004). Thermal Analysis of Plastics: Theory and Practice.

[42] Tu W., Knapik-Kowalczuk J., Chmiel K., Paluch M. (2019). Glass Transition Dynamics and Physical Stability of Amorphous Griseofulvin in Binary Mixtures with Low-Tg Excipients. Mol. Pharm..

[43] Winck J., Daalmann M., Berghaus A., Thommes M. (2022). In-line monitoring of solid dispersion preparation in small scale extrusion based on UV-vis spectroscopy. Pharm. Dev. Technol..

[44] Tao J., Sun Y., Zhang G.G.Z., Yu L. (2009). Solubility of small-molecule crystals in polymers: D-mannitol in PVP, indomethacin in PVP/VA, and nifedipine in PVP/VA. Pharm. Res..

[45] Winck J., Gottschalk T., Thommes M. (2023). Predicting Residence Time and Melt Temperature in Pharmaceutical Hot Melt Extrusion. Pharmaceutics.

[46] Andrews G.P., Qian K., Jacobs E., Jones D.S., Tian Y. (2023). High drug loading nanosized amorphous solid dispersion (NASD) with enhanced in vitro solubility and permeability: Benchmarking conventional ASD. Int. J. Pharm..

